# Melatonin restricts Pb-induced PCD by enhancing BI-1 expression in tobacco suspension cells

**DOI:** 10.1007/s10534-016-9977-6

**Published:** 2016-10-26

**Authors:** A. Kobylińska, Małgorzata M. Posmyk

**Affiliations:** Department of Ecophysiology and Plant Development, Faculty of Biology and Environmental Protection, University of Lodz, 12/16 Banacha Str., 90-237 Lodz, Poland

**Keywords:** Bax inhibitor-1, BI-1, Biostymulators, BY-2 tobacco cells, Melatonin

## Abstract

Melatonin is a conserved substance, which was discovered in the evolutionary distant organisms like bacteria, plants, invertebrates and vertebrates. Recent studies have shown that melatonin despite its possible role in photoperiod processes, has been found to be a direct free radical scavenger and an indirect antioxidant. In this report the impact of exogenous melatonin on the Bax inhibitor-1 (BI-1) expression level in *Nicotiana tabacum* L. line Bright Yellow 2 (BY-2) suspension cells exposed to lead was examined. BI-1 is a well-conserved protein in plants and animals that serves as the inhibitor of mammalian proapoptotic proteins as well as plant ROS-induced cell death. Our results showed that pretreatment with 200 nm melatonin, expressing BI-1 and fortified tobacco suspension cells against damages induced by lead. The obtained results revealed, that melatonin significantly increases BY-2 cells proliferation and protects BY-2 cells against death. Moreover, the conducted analyses showed for the first time that the protective effect of melatonin may be connected not only with its antioxidant properties but also with its direct impact on elevating BI-1 expression and lead-induced programmed cell death (PCD) restriction.

## Introduction

Environment degradation caused by rapid industrial expansion e.g. mining, power generation, transport and intensive agriculture using a lot of chemicals, has become a major threat to the sustenance and welfare of mankind. Heavy metals, among them highly phytotoxic lead (Pb), are major environmental contaminations (Nicholls and Mal 2003).

Lead is present in the atmosphere as dust, fumes, mists, vapors and in soil as minerals (PbCO, PbS, PbSO_4_), so its uptake and accumulation in plants occur either directly, via roots along with water, or it could be absorbed from air via shoots and foliage (Fahr et al. 2013). Unfortunately, plant roots absorb lead with other minerals and then accumulate it in tissues. In several species, a higher lead level causes plant abnormal morphology, reduces plant growth and finally induces cell death (Pourrut et al. 2012).

Pb-provoked growth inhibition and biomass reduction result from heavy metal harmful effects on various plant metabolic processes. Toxic concentrations of this metal disturb or even inhibit activities of key enzymes e.g. acid phosphatase, esterases, peroxidases, malic dehydrogenase, by reacting with their sulfhydril groups. Moreover, it causes water imbalance, alterations in membrane permeability and limits mineral nutrition. High lead concentrations also encourage reactive oxygen species (ROS) generation, thus induces oxidative stress in tissues. Simultaneously, lead induce gradual glutathione depletion as a consequence of cell membrane alteration, DNA damage, gene mutation, protein oxidation, lipid peroxidation and finally provoke signal transduction cascade towards cell death (Wierzbicka 1999; Gill 2014).

Environmental stresses are core factors that limit the biological potential of cultivars and thus decrease plant production. The best solution of this problem seems to be searching and using natural substances—biostymulators, which are able to enhance plant tolerance or protect them against various stresses. Among the different protective substances occurring in plants, melatonin (*N*-acetyl-5-methoxytryptamine) seems to have great biostimulatory potential (Janas and Posmyk 2013). In plants, melatonin is synthesized from l-tryptophan similarly as typical, popular auxin: indole-3-acetic acid (IAA) (Posmyk and Janas 2009). Physiological functions of melatonin have been well documented in animals, although they are currently intensively investigated in plants, melatonin role in the latter one still needs explanation. Melatonin may participate in the regulation of photoperiodic and rhythmic phenomena in plants (Kolář et al. 1997; Tal et al. 2011), it can exhibit auxin-like properties thus influences vegetative development (Arnao and Hernández-Ruiz 2009). Lately, it is pointed that melatonin plays an important role in plant stress defence. Various plant species rich in this indoleamine have shown higher capacity for stress tolerance (Park et al. 2013; Bajwa et al. 2014; Zhang et al. 2015). Melatonin is also involved in stress-affected developmental transitions including flowering, fruiting, ripening and senescence (Kolar et al. 2003; Arnao and Hernández-Ruiz 2009; Zhao et al. 2013; Byeon and Back 2014).

Various stresses inhibit plant growth via different mechanisms but all cause rises in reactive oxygen species (ROS) production and disturb red-ox homeostasis. It is well known that oxidative stress is a secondary effect of all biotic and abiotic ones. Since melatonin has amphiphilic character it may act as hydrophilic and hydrophobic antioxidant. This fact together with melatonin small size makes it particularly able to migrate easily between cell compartments in order to protect them against excessive ROS. Moreover, recent evidence indicates that the melatonin metabolites (Kolodziejczyk et al. 2015), e.g. cyclic-3-hydroxymelatonin, 2-hydroxylmelatonin and especially N1-acetyl-N2-formyl-5-methoxykynuramine (AFMK) also posses antioxidant abilities. It is documented that the free radical scavenging capacity of melatonin extends to its secondary, tertiary and quaternary metabolites (Tan et al. 2000, 2002, 2007a). This phenomenon is referred as the free radical scavenging cascade, and makes melatonin much more efficient even at low concentrations. Thus, melatonin is a broad-spectrum antioxidant and some published data have indicated that even is a far more powerful than C, E, and K vitamins **(**Martinez-Cruz et al. 2002). It seems that evolutionary the strong antioxidant properties of melatonin (Terrón et al. 2001; Maldonado et al. 2007) were its primary role in the defence against unfavourable conditions and in plant stresses tolerance.

In many studies melatonin was observed to reduce oxidative damage of important molecules such as nucleic acids, proteins and lipids (Sliwinski et al. 2007; Posmyk et al. 2009b; Zhao et al. 2011). Its antioxidant activity seems to function via a number of means: (i) as a direct free radical scavenger, (ii) stimulating antioxidant enzymes, (iii) stimulating the synthesis of glutathione, (iv) due to its ability to augment the activities of other antioxidants (v) protecting antioxidant enzymes from oxidative damage (vi) increasing the efficiency of mitochondrial electron transport chain thereby lowering electron leakage and thus reducing free radical generation (Kladna et al. 2003; Rodriguez et al. 2004; Leon et al. 2005; Tan et al. 2007a).

Relatively little is known about the mechanisms of action of melatonin on cytological level in plants. Some information appeared that pretreatment with melatonin attenuated apoptosis in a cold-stressed carrot cell suspension (Lei et al. 2004a).

It is well known that, significant imbalance in cell redox status and oxidative injuries promoted by heavy metals (including lead) can increase cytosolic free calcium ion (Ca^2+^) concentrations which is eventually followed by cell death in aquatic, terrestrial plants as well as in cultured tobacco cells. The induction of cell death by ROS-trigger oxidative stress is often preceded by influx of Ca^2+^. Unfortunately, mechanisms involved in plant cell death are less documented than those in animals. However, ultra structural, physiological and biochemical studies show that plant and animal programmed cell death (PCD) mechanisms share numerous features. These include chromatin condensation, nuclear DNA fragmentation, involvement of ROS, and participation of similar proteins *i.a.* Bax inhibitor-1 (BI-1). Although no gene homologues coding BCL2-associated X protein (BAX) have been identified in plant genomes, Xu and Reed (1998) isolated a mammalian gene of BI-1 that suppressed BAX-induced cell death in yeast. BI-1 is an evolutionary conserved, endoplasmic reticulum-resident protein that represents an ancient cell death regulator that potentially regulates PCD in all eukaryotes. As endoplasmic reticulum (ER) stress signaling pathways have been suggested to play important roles not only in the control of ER homeostasis but also in other biological processes such as the response to pathogens and abiotic stress in plants, BI-1 might function as a swich-factor controlling the convergence point that modulates the level of ‘pro-survival’ and ‘pro-death’ signals under multiple stress conditions (Watanabe and Lam 2009).

Overexpression of BI-1 results in protection against apoptosis induced by certain types of stimuli in mammalian cells, whereas downregulation of BI-1 by an antisense construct promotes apoptosis of some tumour lines (Xu and Reed 1998). Moreover, overexpression of plant BI-1 homologues from *Arabidopsis* and rice (Kawai et al. 1999) can also suppress the BAX-mediated cell death in yeast. Furthermore, AtBI-1 overexpression in *Arabidopsis* inhibits the BAX-mediated cell death in planta (Kawai-Yamada et al. 2001, 2004). Downregulation of BI-1 in cultured rice cells upon challenge with a fungal elicitor from the rice blast pathogen (*Magnaporthe grisea*) was concomitant with progression of cell death, and conversely, overexpressed rice BI-1 can improve cell survival against this elicitor (Matsumura et al. 2003; Wang et al. 2012). BI-1 regulates ROS generation and functions as a Ca^2+^/H^+^ antiporter thus the signaling process of Ca ^2+^ flowing into cytosolic space via ROS-dependent activation of calcium channels on the vacuolar and/or plasma membranes may be controlled by BI-1.

In the present work we studied the effect of melatonin on BI-1 protein expression after tobacco suspension cells exposition to lead stress. We found significant increasing in cells viability and this beneficial effect of exogenous melatonin on Pb-exposed BY-2 cells was correlated with drastically decreasing in H_2_O_2_ concentration and lipid peroxidation but also with a change in the expression of BI-1 protein level—an accepted regulator of plant cell death.

This study aims to provide new insights into differential responses of *Nicotiana tabacum* L. cv. Bright Yellow 2 (BY-2) growing under Pb-stress and explain the biochemical bases of melatonin-induced plant resistance to heavy metals. Taking up the theme of evaluation the beneficial effect of exogenous melatonin on Pb-exposed BY-2 cells we did not expect that increase in cell stress resistance will be connected with a change in the expression level of BI-1 protein—an accepted regulator of plant cell death.

## Materials and methods

### Plant material

In experiments sterile suspension in vito cell culture of *Nicotiana tabacum,* L. cv Bright Yellow 2 (BY 2) were used. Tobacco BY 2 cells were cultivated in Linsmaier and Skoog basal medium (LS) (Linsmaier and Skoog 1965) supplemented with 30 g l^−1^ sucrose, 0.2 mg l^−1^ 2,4-dichlorophenoxyacetic acid (2,4-d; synthetic auxin), 1 mg l^−1^ thiamine, 0.1 g l^−1^ myoinositol and 10^−2^ M KH_2_PO_4_. The initial pH of the medium was established as 5.3.

### Experimental treatments

BY-2 cells of the base culture at the stationary growth phase (day 7th) were passaged into the fresh LS medium as a control (C) and LS with melatonin (MEL) its final concentration in medium: 200 nM. The optimal dose of melatonin was chosen experimentally. In the middle of logarithmic phase of growth (day 4th) Pb(NO_3_)_2_ was added to LS (Pb) and LS with melatonin (MEL + Pb) media to the final Pb^2+^ concentration 15 µM. Thus, experiments were performed in the following variants: (i)** C**: BY-2 cells cultured on LS medium; (ii)** MEL**: BY-2 cells cultured on LS medium with melatonin added from the beginning of culture; (iii)** Pb**: BY-2 cells cultured on LS medium with Pb^2+^ added in the 4th day of culture and (iv)** MEL + Pb**: BY-2 cells cultured on LS medium with melatonin added from the start of culture and stressed with Pb^2+^ added in the 4th day of culture. The cultures were maintained to the 7th day (stationary phase of the control cells growth). The applied concentration of lead was chosen after measurement of LC_50_ at the 7th day.

### Determination of cell growth and viability

The cell number was determined with the use of a Fuchs-Rosenthal haemocytometer under a light microscope; additionally the number of dead cells was assessed after selective staining with methylene blue. The number of cells and their viability were analysed every experimental day.

Morphology of cells was examined in an Olimpus CX-31 light microscope equipped with MicroScan v.15. digital system of image analysis. To detect the morphological changes in all analysed variants, the methylene blue staining method was used. Living cells do not take up the stain and retain their natural colour whereas damaged cells are stained blue as they are unable to keep the methylene blue from penetrating their membranes.

### H_2_O_2_ determination and in situ visualization

Hydrogen peroxide was measured by the method described by Chakrabarty et al. (2009) with slight modifications. Tobacco BY-2 cells were ground in 0.1 % trichloroacetic acid (TCA) (5 cm^3^ g^−1^ cells). After centrifugation, hydrogen peroxide content was measured spectophotometrically after reaction with potassium iodide (KI). The reaction mixture consisted of 0.5 ml of cell extract in 0.1 % trichloroacetic acid (TCA), 0.5 ml of 100 mM K-phosphate buffer pH 7.6 and 2 ml of 1 M KI in fresh deionized water. The blank probe consisted of 0.1 % TCA in the absence of cell extract. After 1 h of reaction in darkness, the absorbance was measured at 390 nm. The amount of hydrogen peroxide was calculated using a standard curve prepared with known concentration of H_2_O_2_ and expressed in nmol g^−1^ fresh weight.

In situ generation of H_2_O_2_ was detected by formation of brown precipitates after incubation of the cells with a solution of 1 mg cm^−3^ 3,3′-diaminobenzidine-tetrahydrochloride (DAB) (Sigma-Aldrich) for 5 h in the light and room temperature. Cells were analyzed under a light microscope Olimpus CX-31 equipped with MicroScan v.15. digital system of image analysis.

### TBARS measurement

Thiobarbituric acid reactive substances (TBARS) are a group of various substances formed as byproducts of lipid peroxidation. They are assayed in tissues for oxidative injury estimation.

The cells (0.5 g) in all experimental variants, were homogenized in 5 ml of 1 % trichloroacetic (TCA) acid and the homogenate was centrifuged at 15,000×*g* for 15 min at 4 °C. The reaction mixture contained: 1 mL of the supernatant and 4 mL of 0.5 % thiobarbituric acid (TBA) in 20 % TCA. In the reference sample, instead of the supernatant, 1 mL of 1 % TCA was added. The mixture was heated at 95 °C for 30 min in a water bath and then cooled in an ice bath. After centrifugation, the absorbance was measured at 532 nm in UV/vis spectrophotometer (Hitachi U-2001, Hitachi Instruments Inc., Japan). The value for non-specific absorption at 600 nm was subtracted (Heath and Parker 1968). The TBARS content was calculated according to MDA extinction coefficient of 155 mM^−1^ cm^−1^ and expressed as µmol MDA per g of FW. The TBARS content was measured in the end of experiment (day 7th) for estimation of potential membrane injuries provoked by lead-stress and/or alleviated by melatonin application.

### Total phenolic concentration

Phenolic compounds were extracted with pure methanol from all experimental variants. After centrifugation (1500×*g*, 10 min) BY-2 suspension cultures were re-suspended in methanol (10 ml methanol/0.2 g cells) and kept in darkness for 24 h on a rotary shaker 100 rpm at room temperature.

After next centrifugation (1500×*g*, 10 min) the supernatants were collected. Total phenolic content was estimated by the Folin-Ciocalteu method using gallic acid as a standard (Slincard and Singleton 1977). 50 μl of the extract were combined with 1.55 ml of distilled water, 100 μl of Folin-Ciocalteu’s reagent and 300 μl of 20 % Na_2_CO_3_. The mixture was vortexed thoroughly and, after incubation at 40 °C for 30 min, the absorbance was measured at 765 nm against a ‘blank’ without the sample extract. Quantification was done on the basis of the standard curve of gallic acid (solution 0.25–5 μg ml^−1^).

Total phenolic content was measured: (i) during *lag* phase (1st day), when the cells were transferred to the new medium and they adapted to new growth conditions, (ii) during the logarithmic phase of growth (*log* phase) (day 4th after passaging), when the number of new cells appearing per unit time was proportional to the present population, this is a period of intensive proliferation, (iii) during stationary phase of growth (day 7th) when the number of newly created cells was limited by the growth factor and as a result the rate of cell growth matched the rate of cell death. The results were expressed as mg of gallic acid equivalents (GAE) per g of cells.

### Melatonin determinations

Melatonin was extracted according to the modified methods of Guerrero et al. (2001) and Hernandez-Ruiz et al. (2004) with modifications. The concentrations of melatonin were determined in BY-2 cell extracts using high-performance liquid chromatography (HPLC–MS). For extraction, the cells (5 g fresh weight) were homogenized with 5 mL of 50 mm sodium phosphate buffer (pH 8.0) containing 1 mm EDTA and 5 μM butylated hydroxytoluene (BHT) as antioxidant. The homogenate was maintained for 15 h at room temperature in darkness with minimal shaking, in order to ensure complete extraction of melatonin.

Afterwards, it was centrifuged at 15,000×*g* for 10 min at 5 °C. Initial purification consisted in two steps by solvent-partitioning using ethyl acetate and 50 mm sodium phosphate buffer (first at pH 8.0 and second at pH 3.0). The two organic phases were evaporated together under vacuum. Dry residue was re-dissolved in 1 mL of mobile phase, filtered through Supelco ISO-Disc filters (PTEF-4–2.4 mm × 0.2 m; Supelko, Bellefonte, PA, USA), and frozen at −70 °C until HPLC–MS analysis. The purified extract was subjected to HPLC–MS/MS analysis using an Agilent 1200 LC System coupled with AB Sciex 3200 QTRAP mass detector equipped with TurboSpray Ion Source (ESI). Each sample was injected onto Agilent SB-C18 column. Melatonin concentration was measured analogously to phenolic compounds: during *lag, log* and the stationary phases of growth.

### Cell lysates and western blot analysis

BY-2 cell lysates were prepared to assess the expression of BI-1 protein: BY-2 cells were lysed (4 °C; 20 min) in a buffer containing an enzyme cocktail for cell wall lysis (CellLytic Sigma), 10 mM Tris–HCl (pH 7.5), 300 mM NaCl, 1 % Triton X-100, 2 mM MgCl_2_, 0.1 M DTT, and phenylmethylsulfonyl fluoride to a final concentration of 1 mM. After centrifugation at 10,000×*g* for 15 min, the supernatants were collected. Protein content was estimated by the method of Lowry et al. (1951). The lysates (50 μg of proteins) were electrophoretically separated by sodium dodecyl sulfate polyacrylamide gel electrophoresis (SDS-PAGE) on 11.2 gel (Laemmli 1970) and transferred to Immobilon P according to Towbin et al. (1979). After blocking in 3 % nonfat dry milk and TBS (10 mM Tris–HCl, pH 7.5, 150 mM NaCl) for 60 min, the membranes were incubated with antibodies specific to BI-1 in TBS in a cold room overnight. Rabbit polyclonal antibodies (used at appropriate dilution) were purchased from Santa Cruz Biotechnology (USA). Subsequently, the membranes were washed several times in TBS containing 0.05 % Tween-20 (TBST), and incubated with appropriate secondary antibodies conjugated with alkaline phosphatase (Sigma Chemical Co.) in TBS for 2 h at room temperature. Next the membranes were washed several times with TBST, and the proteins were visualized by incubation with substrate solution (0.33 mg/ml of nitro blue tetrazolium, 0.17 mg/ml of 5-bromo-4-chloro-3-indolyl phosphate in 100 mM Tris–HCl, pH 9.5, 100 mM NaCl and 5 mM MgCl_2_), prepared according to Leary et al. (1983).

### Statistical analyses

The data represent the mean ± standard deviation (±SD). Each variant of culture was replicated 3 times and up to three independent samples were used for measurement. The data were analysed using STATISTICA v.10.0_MR1_PL [StatSoft] software. One-way or two-way analysis of variance (ANOVA) and then the post hoc Duncan multiple range test was carried out to find the significant differences at p < 0.001 in each experiment.

## Results

### Cell growth and viability after lead treatment

Preincubation with melatonin prior to lead treatment protected tobacco suspension cells from dying and improve cell proliferation.

**Fig. 1:**
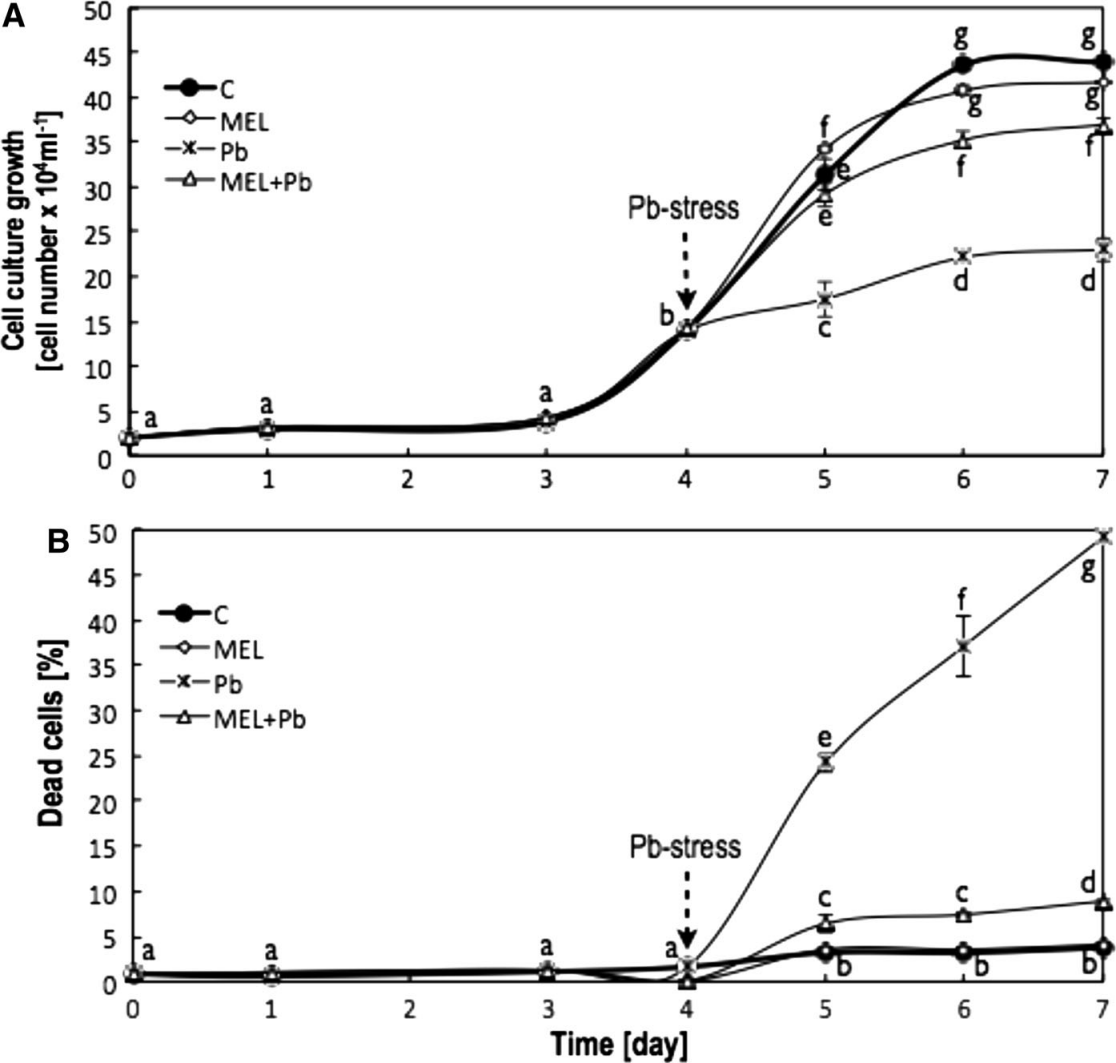
The kinetics of changes in proliferation level (**a**) and mortality (**b**) of BY-2 tobacco cells in conducted experiments.** C**—BY-2 cells cultured on LS medium—control variant;** MEL**—BY-2 cells cultured on LS medium with 200 nM melatonin added from the beginning of culture;** Pb**—BY-2 cells cultured on LS medium with 15 µM Pb^2+^ added in the 4th day of culture and** MEL + Pb**—BY-2 cells cultured on LS medium with melatonin added from the start of culture and with Pb^2+^ added in the 4th day of culture. On both graphs, for variants Pb and MEL + Pb, start time of Pb-stress is marked by *arrow*. The cultures were maintained to the 7th day—stationary phase of the control cells growth. The results are expressed as mean values of 3 independent experiments ± SD. Two-way ANOVA and Duncan’s post hoc test were performed. The small letters next to the values show statistical significance p < 0.001. **a** Viability ANOVA results: Variant (C, MEL, Pb, MEL + Pb) F_(3;56)_ = 1189 p < 0.0001; Time (0, 1, 3, 4, 5, 6, 7) F_(6;56)_ = 11505 p < 0.0001; and interaction Variant x Time F_(18;56)_ = 270 p < 0.0001. **b** Mortality ANOVA results: Variant (C, MEL, Pb, MEL + Pb) F_(3;56)_ = 3207 p < 0.0001; Time (0, 1, 3, 4, 5, 6, 7) F_(6;56)_ = 1637 p < 0.0001; and interaction Variant x Time F_(18;56)_ = 756 p < 0.0001

Growth rates of the C and MEL cells were similar during all culture time. Pb-stress started on 4th day resulted in significant inhibition of tobacco cell proliferation—see variants MEL + Pb and especially Pb (Fig. 1a). However, from the 1st day after lead treatment, proliferation of the MEL + Pb cells was about 40 % higher in comparison to those, Pb-treated and not primed with melatonin ones-Pb (Fig. 1a). This effect persisted for the entire duration of the BY-2 cell variants subjected to Pb-stress.

Next, the protective role of melatonin was evaluated by cell mortality assessment in all experimental samples. Methylene blue staining evidenced the inhibitory effect of melatonin on cell death induced by lead.

Culture medium supplementation with melatonin did not result in cell death acceleration (slight differences comparing with control was not statistically important). Expectedly, the number of dead cells in Pb variant increased significantly during heavy metal stress and was: 24.2, 37.3 and 49.2 % for the 5th, 6th and 7th day (i.e. 1st, 2nd and 3rd day after lead application) respectively (Fig. 1b). In contrast, in the melatonin pre-incubated variant exposed to heavy metal (MEL + Pb), cell viability was about 80 % higher than in the Pb cells (Fig. 1b).

### Determination of melatonin levels in BY-2 cells

To find out whether melatonin treatment results in its level increase in BY-2 cells, i.e. whether tobacco cells are able to active melatonin absorption, the contents of this indoleamine in cell lysates were determined by HPLC–MS in curtail points of experiments.

**Fig. 2:**
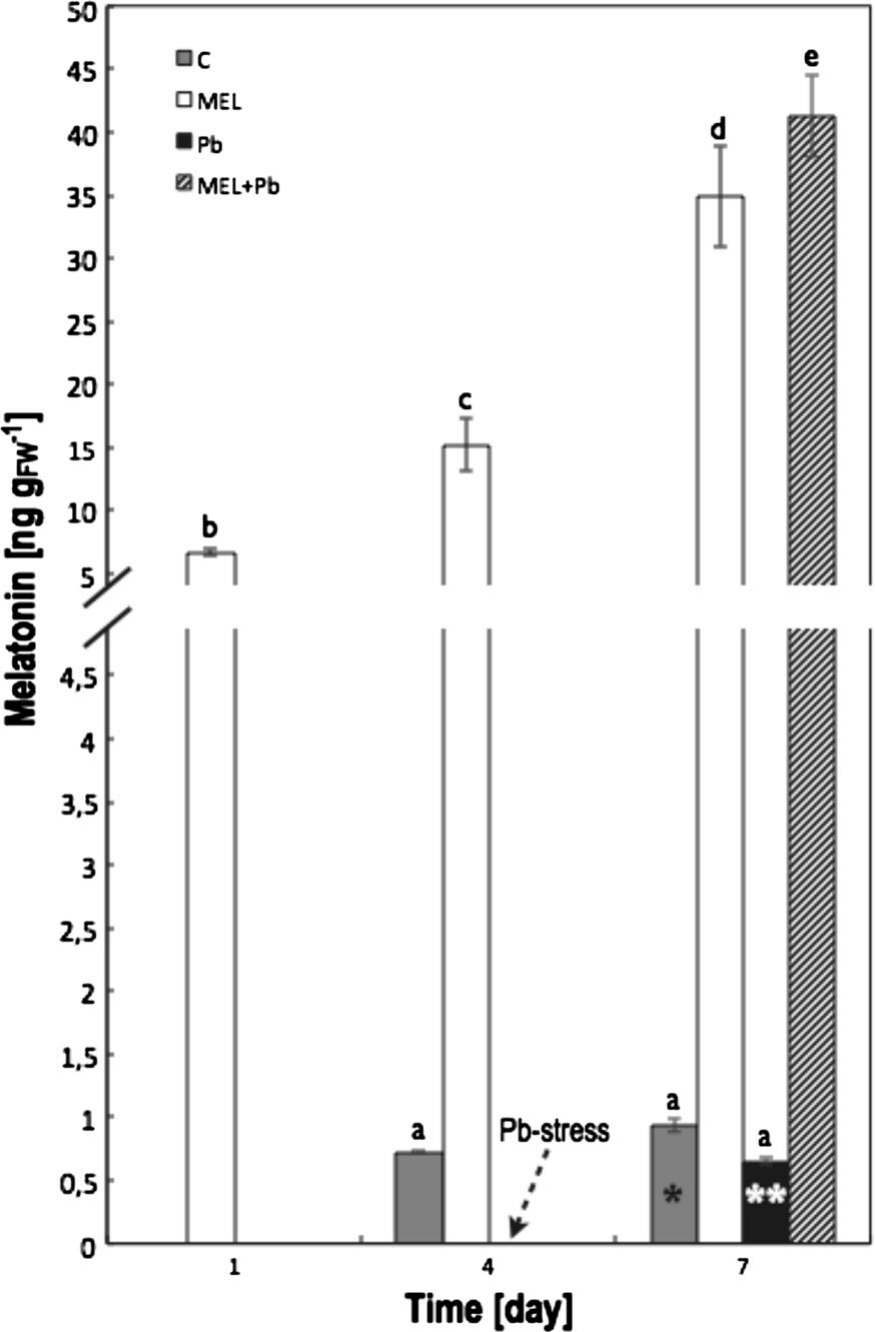
Melatonin determination in BY-2 tobacco cells in crucial points of conducted experiments. HPLC–MS measurements was performed: 1st day—during *lag* phase; 4th day after passaging—during *log* phase, this day was also chosen as the start of Pb-stress (*marked by arrow*); and 7th day—during stationary phase of growth (variants C and MEL) and when Pb-stress symptoms should be detectible (variants Pb and MEL + Pb). Experimental BY-2 cell variants:** C**—culture on LS medium—control variant;** MEL**—culture on LS medium with 200 nM melatonin;** Pb**—LS medium culture treated with 15 µM Pb^2+^ added in the 4th day; and** MEL + Pb**—culture on LS medium with melatonin treated with Pb^2+^ added in the 4th day. On both graphs, for variants Pb and MEL + Pb, start time of Pb-stress is marked by *arrow*. The cultures were maintained to the 7th day—stationary phase of the control cells growth. The results are expressed as mean values of 3–4 measurements ± SD. Two-way ANOVA and Duncan’s post hoc test were performed. The small letters next to the values show statistical significance p < 0.0001. Melatonin ANOVA results: Variant (C, MEL, Pb, MEL + Pb) F_(3;24)_ = 440 p < 0.0001; Time (1, 4, 7) F_(2;24)_ = 317 p < 0.0001; and interaction Variant x Time F_(6;24)_ = 100 p < 0.0001. In a case of 7th day C and Pb variant additionally Student’s *t*-test were performed and the statistically significant difference between this two values was determined at *p* < 0.05 (*marked by stars*)

Generally, BY-2 tobacco cells are deficient in melatonin content (Fig. 2—low part of scale). In non melatonin-primed cells: C and Pb its level slightly increased from zero (initial culture day) to ~1 ng/g_FW_ (on the last day). These cells are able to synthesize endogenous melatonin, but its level remained extremely low in comparison with the melatonin-primed cell variants: MEL and MEL + Pb (Fig. 2—high part of scale). In addition, lead treatment (Pb) resulted in diminished endogenous level of this indoleamine by about 30 % in comparison to control cells (C) at the end of culture period (7th day). This differences was statistically confirmed only at *p* < 0.05 by additionally performed Student’s *t* test compared only this two seed variants (C and Pb on 7th day).

The concentrations of melatonin significant increased in cells during the whole period of melatonin treatment (Fig. 2—high part of scale). It means that tobacco cells absorbed it progressively from medium. Interestingly, Pb-treated cells (MEL + Pb) absorbed it more intensively in comparison to unstressed (MEL) ones. The obtained results indicated that the level of melatonin in MEL + Pb variant was about 20 % higher than in MEL cells.

### Content and in situ accumulation of hydrogen peroxide

**Fig. 3:**
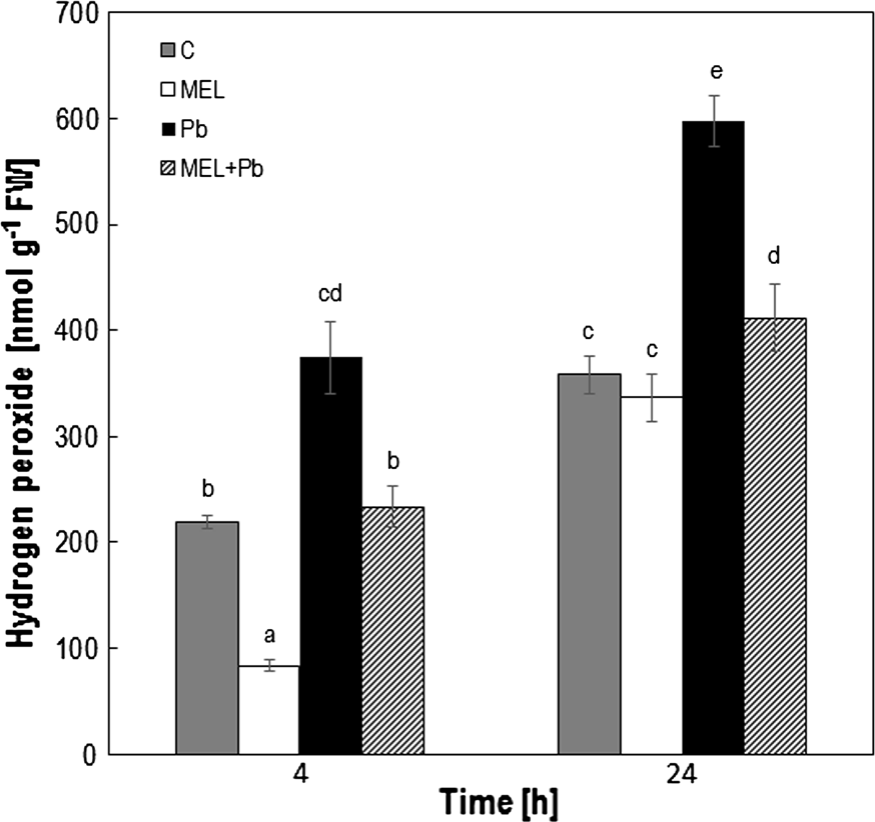
Hydrogen peroxide concentration in tobacco BY-2 cells. Measurements ware performed 4, and 24 h after lead treatment. Experimental BY-2 cell variants: C—culture on LS medium—control variant; MEL—culture on LS medium with 200 nM melatonin; Pb—LS medium culture treated with 15 µM Pb^2+^ added in the 4th day; and MEL + Pb—culture on LS medium with melatonin treated with Pb^2+^ added in the 4th day. The results are expressed as mean values of 6–9 measurements ± SD. Two-way ANOVA and Duncan’s post hoc test were performed. The small letters next to the values show statistical significance p < 0.0001. Melatonin ANOVA results: Variant (C, MEL, Pb, MEL + Pb) F_(3;53)_ = 106 p < 0.0001; Time (4 h, 24 h) F_(1;53)_ = 277 p < 0.0001; and interaction Variant x Time F_(3;24)_ = 3.9 p < 0.01

DAB staining used for microscopy detection of H_2_O_2_ was carried out following biochemical quantitative analyzes. Extremely low H_2_O_2_ concentration was observed at the beginning of *log* phase of growth in MEL cells and it was continued till the 4th day. In the middle of *log* phase (the 5th day of culture; 24 h after lead treatment) concentration of H_2_O_2_ was similar to control. The H_2_O_2_ concentrations 4 h and 24 h after lead exposition in only Pb-treated cells was 70 % higher than in the control and reached the maximal level near 600 nmol g^−1^ FW in the second day after lead treatment (the 5th day after start the new culture). Interestingly, in BY-2 cells treated with heavy metal but primed with melatonin (MEL + Pb) the H_2_O_2_ concentration was 40 % and near 60 % lower than in Pb-cell at 4 and 24 h after lead application, respectively (Fig. 3).

**Fig. 4:**
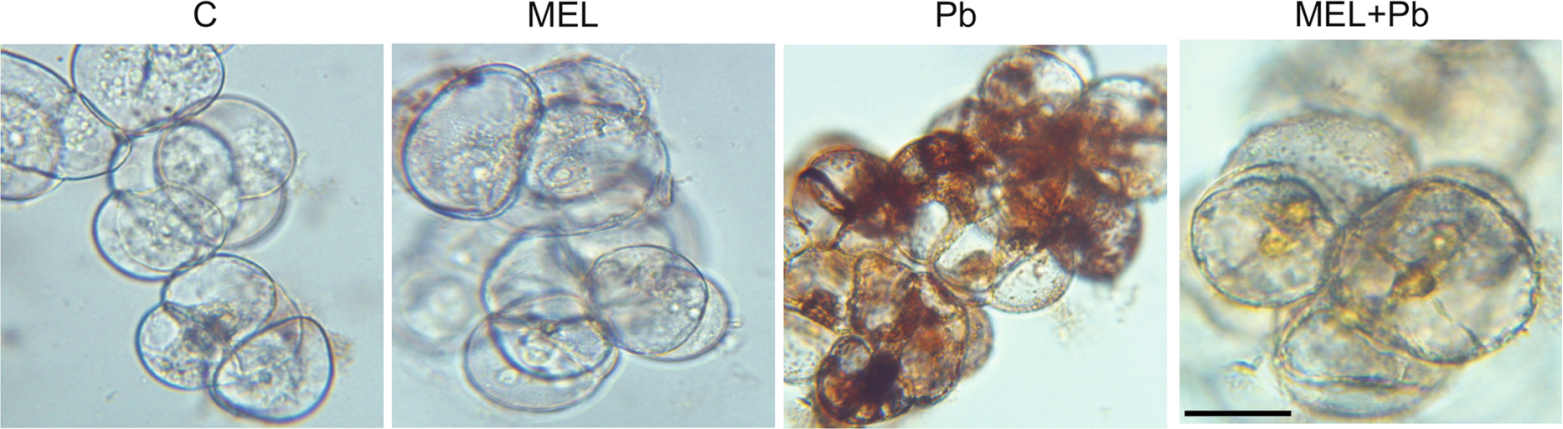
Hydrogen peroxide in BY-2 cells visualised by DAB staining 24 h after lead treatment (it corresponds to the results presented in Fig. 3). Experimental BY-2 cell variants: C—culture on LS medium—control variant; MEL—culture on LS medium with 200 nM melatonin; Pb—LS medium culture treated with 15 µM Pb^2+^ added in the 4th day; and MEL + Pb—culture on LS medium with melatonin treated with Pb^2+^ added in the 4th day. Bar = 40 µm

*In situ* staining of H_2_O_2_ production in tobacco suspension cells and cytological analyses demonstrated, many brown deposits in Pb variant. They were especially visible in the boundary cytoplasm and in nuclei (Fig. 4). In contrast, in the MEL + Pb samples the amounts of precipitants was near similar to that in the control, confirming antioxidant properties of melatonin.

### Effect of melatonin on TBARS and total phenolic content after lead application

In the end of experiment (day 7th)—after 3 days of Pb-treatment TBARS content was measured in all experimental variants to estimate membrane oxidation/injuries provoked by lead-stress and/or alleviated by melatonin application. In the stationary phase (day 7th), when gradual increase in the dead cell number due to the nutrient consumption was observed, TBARS content was about 23 and 13 % lower for MEL + Pb versus Pb and MEL versus C, respectively (Table 1). Thus these biochemical and cytological observations demonstrated clearly that melatonin pre-incubation protected tobacco suspension cells from lead-induced lipid peroxidation and TBARS accumulation.

**Table 1:** TBARS determination in BY-2 tobacco cells in the end of experiments—on 7th day, during stationary phase of growth (variants C and MEL) and when Pb-stress injuries should be detectible (variants Pb and MEL + Pb)

BY-2 cell variant	TBARS [mmol ${\mathbf{g}}_{\text{FW}}^{ - 1}$]	±SD
C	2.45*b*	0.25
MEL	2.13*a*	0.06
Pb	2.76*c*	0.29
MEL + Pb	2.14*a*	0.15

Experimental BY-2 cell variants: **C**—culture on LS medium—control variant;** MEL**—culture on LS medium with 200 nM melatonin;** Pb**—LS medium culture treated with 15 µM Pb^2+^ added in the 4th day; and** MEL + Pb**—culture on LS medium with melatonin treated with Pb^2+^ added in the 4th day. In botch Pb-treated variants (Pb and MEL + Pb) stress duration was last 3 days. The results are expressed as mean values of 7–9 measurements (*n* = 7–9) ± SD. One-way ANOVA and Duncan post hoc test were performed. The small letters next to the values show statistical significance p < 0.0001. ANOVA results: Variants (C, MEL, Pb, MEL + Pb) F_(3;25)_ = 15.5 p < 0.0001

The next step of experiments was to evaluate whether protective effect of melatonin can be additionally mediated by phenolic compounds accumulation. It is well known, that secondary metabolites, including polyphenols can be synthesized de novo in response to biotic and abiotic stresses and perform an antioxidative function (Dixon and Paiva 1995; Janas et al. 2009).

**Fig. 5:**
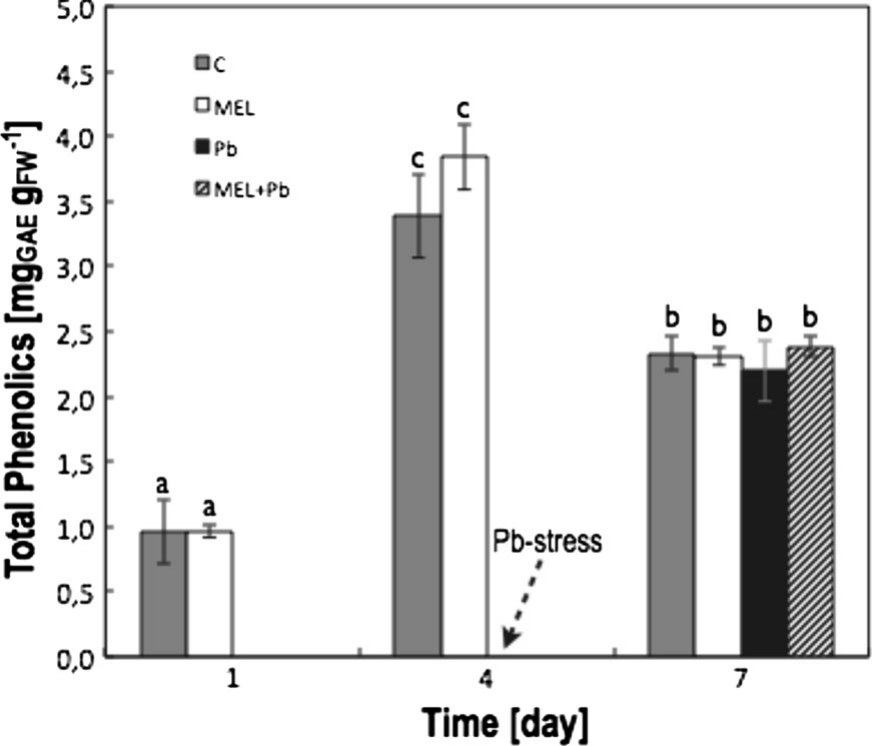
Total phenolic compounds determination in BY-2 tobacco cells in crucial points of conducted experiments. Measurements was performed: 1st day—during *lag* phase; 4th day after passaging—during *log* phase, this day was also chosen as the start of Pb-stress (marked by *arrow*); and 7th day—during stationary phase of growth (variants C and MEL) and when Pb-stress symptoms should be detectible (variants Pb and MEL + Pb). Experimental BY-2 cell variants: **C**—culture on LS medium—control variant;** MEL**—culture on LS medium with 200 nM melatonin;** Pb**—LS medium culture treated with 15 µM Pb^2+^ added in the 4th day; and** MEL + Pb**—culture on LS medium with melatonin treated with Pb^2+^ added in the 4th day. On both graphs, for variants Pb and MEL + Pb, start time of Pb-stress is *marked by arrow*. The cultures were maintained to the 7th day—stationary phase of the control cells growth. The results are expressed as mean values of 7–9 measurements ± SD. Two-way ANOVA and Duncan’s post hoc test were performed. The small letters next to the values show statistical significance p < 0.01. Phenolics ANOVA results:** Variant** (C, MEL, Pb, MEL + Pb) F_(3;86)_ = 1.82** p = 0.150** (differences between variants were not statistically significant); Time (1, 4, 7) F_(2;86)_ = 671.6 p < 0.0001; and interaction Variant x Time F_(6;86)_ = 3.06; p < 0.01

Low level of phenolics content in the initial phase of growth (1st day) significantly increased to 4th day after passaging: 3.5 and 4 times during the logarithmic phase of growth in C and MEL variant respectively (Fig. 5). Unfortunately, during stationary phase of cell growth (day 7th) phenolics content decreased to ~70 %. Lead stress from 4th day of culture intensified this decrease, but melatonin application prior and simultaneously with stress seems to slightly alleviated this tendency—unfortunately two-way analysis of variance (ANOVA) did not confirm statistical importance as concern differences between variants (Fig. 5).

### Influence of melatonin on BI-1 protein expression after lead application

**Fig. 6:**
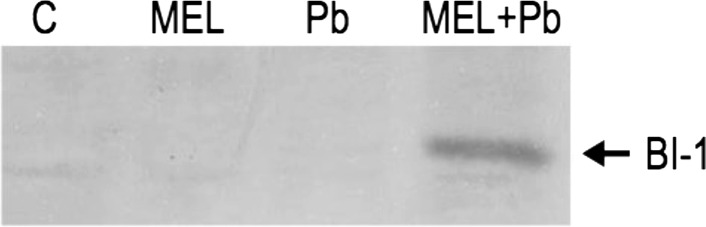
Expression of BI-1 protein on stationary phase of cell growth (7th day). Lysates from untreated BY-2 cells (C), cells grown on medium supplemented with melatonin (MEL) and cells exposed to lead without and with melatonin treatment (Pb and MEL + Pb, respectively)

**Fig. 7:**
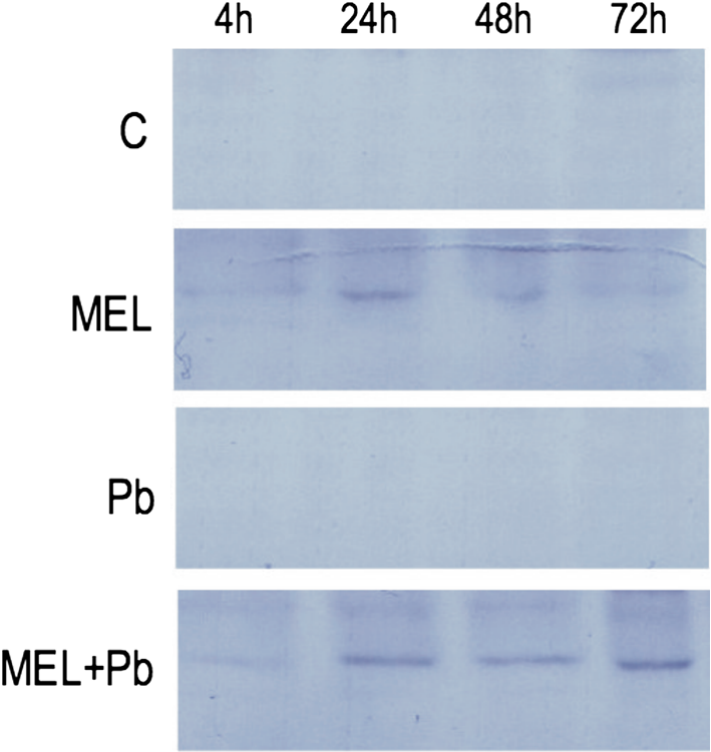
Expression of the BI-1 protein 4, 24, 48 and 72 h after lead treatment. Lysates from untreated BY-2 cells (C), cells grown on medium supplemented with melatonin (MEL) and cells exposed to lead without and with melatonin treatment (Pb and MEL + Pb, respectively)

Taking into account that in the last stages of culture (from 4th to 7th day) cells pretreated with melatonin and then subjected to Pb-stress (MEL + Pb) showed much higher proliferation ability (Fig. 1a) and especially lower mortality (Fig. 1b) in comparison to not protected by melatonin, only stressed ones (Pb) the next part of presented studies was the analysis of BI-1 protein expression, which is one of the ancient death regulators of general importance for cellular homeostasis. Firstly, cell lysates of all experimental cell variants were probed by Western blotting, and expression of BI-1 was examined on 7th day—at the end of culture period (Fig. 4),

The expression of BI-1 both in MEL and Pb cells was very low, almost undetectable and comparable to the control ones (C) (Fig. 4). Only combination of factors: melatonin application prior and during Pb-stress (MEL + Pb) caused surprisingly high level of BI-1 expression (Figs. 6 and 7).

This result was accompanied with drastically lower number of dead cells (Fig. 1b) and TBARS level (Table 1) in MEL + Pb variant in comparison to Pb cells.

Additionally, we verified the BI-1 immunoreactivity in all examined cell variants at 4, 24, 48, 72 h after heavy metal exposition. Figure 5 shows gently immunostained BI-1 in the MEL cells in the middle of the *log* phase of growth. These results suggest that melatonin may act as a factor fortifying cells (Fig. 5, see first 24 h of *log* phase) against potentially stress conditions even before it appears. The significant, gradually increasing BI-1 expression level was determined in MEL + Pb cells from 24 to 78 h after Pb-treatment (5th to 7th day). At the end of the culture it was the strongest.

Its worthy to note, that at the end of culture, outright BI-1 expression was determined only in MEL + Pb samples. Slight symptoms of this protein expression was visible shortly in non stressed MEL cell variant, but in control ones (C) and these, which were dying under stress condition (Pb) BI-1 immunoreactivity was not detected.

## Discussion

Although significance of melatonin for plants is not fully elucidated yet, many researchers underline that among its various roles melatonin’s antioxidant effectiveness and free radical scavenging ability, that protect plants against oxidative stress and alleviate or counteract cell damages, are extremely crucial and similar as in animals.

Melatonin anti-apoptotic action associated with its antioxidant properties is well documented in various animal cells (Reiter et al. 1997; Tian et al. 2001), but not in plants. Generally, signal transduction in PCD processes is still intensively investigated in plants. Due to the differences discovered some scientist suggests that in plants it should be called PCD-like process (Reape and McCabe 2010).

Since endogenous melatonin has been found in a variety of plants its role in stresses tolerance and counteracting is widely propagated i.g.: (i) in various plants subjected to stress endogenous melatonin level increase (Tal et al. 2011); (ii) plants rich in melatonin are less susceptible to different stresses (Park et al. 2013; Bajwa et al. 2014; Zhang et al. 2015) and (iii) exogenous melatonin application act as plant biostimulator especially under harmful environment conditions (Posmyk et al. 2008b, 2009b; Janas and Posmyk 2013; Kołodziejczyk et al. 2016); additionally it could be applied as preventive factor before stress appear and/or as intervention during stress (Kołodziejczyk and Posmyk 2016).

Taking above into account with the fact that melatonin is an important antioxidant and anti-apoptotic factor in animal cells, we would like to investigated its influences on heavy metal induced cell death in tobacco suspension cultures. To our knowledge, this is the first report regarding the protective effect of melatonin on in vitro plant suspension cells exposed to Pb-stress. The selection of the in vitro plant model to tests was dictated by the homogeneity of the material (suspension of undifferentiated cells) and possibility of multiple repetitions of the experiments under the same strictly controlled conditions.

At the first part of this study cell proliferation and viability (culture development in *lag, log* and stationary phase) under optimal (C and MEL) and heavy metal stress condition (Pb and MEL + Pb) were investigated. *Nicotiana tabacum* BY-2 cells cultivation on LS medium supplemented with melatonin (MEL) did not change significantly cell development profiles (Fig. 1ab—proliferation/mortality) characteristic for non-treated control cells cultivated under optimal conditions. Its positive effects were visible during and after Pb-stress. Proliferation of cells pre-treated with melatonin under heavy metal stress condition (MEL + Pb) was narrowly worse then unstressed ones (C and MEL) whereas the same Pb-stress significantly decreased proliferation level (more then 50 %) of control cells—variant Pb (Fig. 1a). The protective role of melatonin was extremely visible comparing cell mortality during Pb-stress. Expectedly, the number of dead cells in Pb variant increased drastically during heavy metal stress, in contrast to melatonin pre-incubated cell variant exposed to heavy metal (MEL + Pb), where their viability was about 80 % higher than in the Pb cells (Fig. 1b). Our results are similar to many previous, which also indicated that positive effects of plant melatonin treatments do not appear under optimal conditions but such a plants are stronger when have to face to stresses (Posmyk et al. 2008b, 2009b). Theses results were in line with studies showing that melatonin reduce ROS level caused by Pb-stress. As it was mentioned in results section, the H_2_O_2_ concentration was much lower in MEL + Pb cells than in Pb ones. Moreover, in situ staining showed that accumulation of H_2_O_2_ in lead-treated cells but primed with melatonin was similar to these in not stressful conditions. Our analyses also demonstrated that this indoloamine alleviated oxidative injuries. In the end of culture (day 7th), when gradual increase in the dead cell number due to the nutrient consumption and/or stress injuries were observed, TBARS content was about 25 and 15 % lower for MEL + Pb versus Pb and MEL versus C, respectively (Table 1).

Many of recent papers have indicated that high melatonin level in plants is positively correlated with their higher capacity for unfavorable conditions tolerance (Park et al. 2013; Tal et al. 2011; Bajwa et al. 2014; Zhang et al. 2015). The importance of melatonin in stress symptoms/disturbances limitation during different plant developmental stages such as flowering, fruiting, and senescence, was widely discussed (Kolar et al. 2003; Arnao and Hernández-Ruiz 2009; Zhao et al. 2013; Byeon and Back 2014). Thus, these biochemical and cytological observations demonstrated clearly that melatonin pre-incubation protected tobacco suspension cells from lead-induced lipid peroxidation and TBARS accumulation. Moreover, our team demonstrated, that melatonin acts not only as a antioxidative agent but also prevents DNA fragmentation (data in press). Similar protective effects caused by exogenous melatonin was observed by Posmyk et al. in red cabbage seedlings subjected to copper stress (Posmyk et al. 2008b) and in cucumber seedlings subjected to chilling stress (Posmyk et al. 2009b).

The plants possess many defense strategies to cope with lead toxicity, including lead reduced uptake, its sequestration into vacuoles and/or cell walls by formation of complexes, Pb-binding by phytochelatins, glutathione and aminoacids, as well as synthesis of osmolytes (Sengar et al. 2008). Moreover, plants may activate various antioxidants constituting defense system against secondary oxidative stress provoked by lead. System is based on cooperation between enzymatic (e.g. SOD, CAT, POX) and non-enzymatic antioxidants such as: ascorbate, glutathione, proline, melatonin and phenolic compounds (e.g. phenolic acids and flavonoids). Phenolic compounds can accumulate at high concentrations as constitutive compounds (Janas et al. 2002; Szafrańska et al. 2013), but they can also be synthesized de novo in response to environmental stresses (Dixon and Paiva 1995; Janas et al. 2009). These compounds play an important role in the control of many biological activities in plants, acting as, e.g. enzyme inhibitors, light-absorbing pigments, light screens, visual attractants for pollinators, regulators of plant growth, chemical signals in nodulation gene induction, as well as phytoalexins (Grace 2005; Szafrańska et al. 2013). Moreover, applying exogenous plant polyphenols would be useful for stress alleviate both in plant and animal tissues (Posmyk et al. 2008a, 2009a; Kolodziejczyk et al. 2011; Saluk et al. 2012, 2015).

Many publications pointed the fact, that positive effects of melatonin could be caused not only by its own antioxidant properties (Reiter et al. 2015), but also by its influence on synthesis/accumulation of other compounds e.g. polyamines (Lei et al. 2004) or phenolics (Janas et al. 2000, 2002) in plant tissues during stress. Thesis that melatonin could additionally influenced phenolic compounds content/biosynthesis in tobacco cells was not confirmed by our results. Generally phenolics content in BY-2 cells fluctuates during culture. It was lowest in the initial phase of growth (1st day) then increased to 4th day after passaging (and then was much higher in MEL than in C) but finally decrease during stationary phase of cell growth (day 7th) (Fig. 3). Lead stress from 4th day of culture intensified this decrease, unfortunately melatonin application prior and simultaneously with stress, only slightly alleviated this tendency (Fig. 3).

However, it should be mentioned that the level of phenolic compounds is not always increased under stress conditions (Płażek et al. 2000; Roitto et al. 2005; Szafrańska et al. 2012). Szafrańska et al. (2012) demonstrated that under chilling stress total phenolic level was not changed but ratio between their particular components might be different.

Our present results indicated that poor in melatonin tobacco BY-2 cells are able to synthetize this indoleamine as well as to absorb it actively from the medium (Fig. 2). Availability of exogenous melatonin causes it to be taken up by BY-2 cells in large quantities. Their levels were 15-40 fold greater in melatonin-treated cells (MEL and MEL + Pb) than in untreated ones (C and Pb) (Fig. 2). The same phenomenon was observed in a case of cucumber and corn seeds that were osmo- or hydroprimed with exogenous melatonin (Kołodziejczyk et al. 2015)—they absorbed great quantities of indoleamine proportionally to its concentration applied during priming. Interestingly in presented results, Pb-stressed cells (MEL + Pb) absorbed melatonin under harmful conditions more intensively in comparison to unstressed (MEL) ones. It could suggest that BY-2 cells to counteract stress-induced damage probably willingly utilize exogenous melatonin.

Plants continuously produce a certain amount of ROS during normal cellular metabolism. They are useful in peroxisomes functioning, during cell wall lignification and lipid beta-oxidations in glyoxysomes and also they work as signaling molecules. However Bolduc and Brisson (2002) as well as Kawai-Yamada et al. (2004) reported that a burst of oxidative metabolism leading to generation of ROS was one of the earliest events in PCD induced by biotic or abiotic stress in tobacco plants. This suggests that high levels of ROS mediate the signal network, on the one hand—for defense gene induction e.g. *hsp*, *lea*, *cor*, -*ecs* (Vincour and Altman 2005), but on the other hand—for PCD induction of selected cells.

Stress response and cell death regulation in plants is not well understood. Many efforts to isolate pro- or anti-apoptotic homologues of the animal Bcl-2 protein family from plants failed. Nevertheless, there are some similarities between plant and animal PCD that indicate common elements in both systems. For instance, reactive oxygen intermediates, cysteine proteases, DNA degradation, and some morphological changes seem to take part in both animal and plant PCD (Wang et al. 2012). Identification of some plant homologues of animal cell death suppressors, for instance BAG (Bcl-2 associated athano- gene), DAD (defender against apoptotic death) and BI-1, indicates common elements of negative cell death control for eukaryotes. BI-1 proteins are highly conserved among humans, animals, and plants. However, the mechanism by which BI-1 inhibits PCD still requires elucidation (Huckelhoven et al. 2003).

Kawai-Yamada et al. (2004) showed, that H_2_O_2_-induced cell death in tobacco BY-2 suspension cells was suppressed by overexpression of BI-1. Thus, BI-1 protein is proposed to be a conserved cell death inhibitor (Kawai-Yamada et al. 2004). Moreover, BI-1 overexpression not only confers tolerance to oxidative stress-mediated cell death but also enhances metabolic acclimation involved in energy and redox balance (Ishikawa et al. 2010).

In our final tests, the expression of BI-1 both in MEL and Pb cells was very low, almost undetectable and comparable to the control ones (C) (Fig. 4). Only combination of factors: melatonin application prior and during Pb-stress (MEL + Pb) caused surprisingly high level of BI-1 expresion (Figs. 4, 5). More precise investigations show gently immunostained BI-1 in MEL non-stressed cells during the middle of the *log* phase of growth too. These results suggest that melatonin may act as a factor fortifying cells (Fig. 5, see first 24 h of *log* phase) against potentially stress conditions even before it appears. The significant, gradually increasing BI-1 expression level was determined in MEL + Pb cells from 24 to 78 h after Pb-treatment and these results were correlated with strong decrease in cell mortality of MEL + Pb cells (Fig. 1b)—what means better viability and higher ability to proliferation under stress conditions (Fig. 1a).

Similar results were published by Huckelhoven et al. (2003), who showed that resistance of barley to the fungus *Blumeria graminis* f. sp. *Hordei (Bgh)* was associated with overexpression of BI-1 in barley epidermal cells during interaction with *Bgh*. Nagano et al. (2012) demonstrated that the cell death suppression by BI-1 was mediated, at least in part, through fatty acid hydroxylase 91 (FAH). In addition, it was reported that *Arabidopsis* FAHs (AtFAH1 and AtFAH2) interacted with BI-1 via cytochrome b5 at ER, resulting in accumulation of 2-hydroxy fatty acids (2-HFAs) in *Arabidopsis* plants overexpressing BI-1 (Ishikawa et al. 2015). These findings indicated that BI-1 altered sphingolipid composition in membranes, and this was accompanied by dynamic changes in a number of plasma membrane microdomain proteins involved in cell death regulation. Although involvement of sphingolipids in plant stress tolerance and cell death regulation is gradually being revealed (Brodersen et al. 2002; Chen et al. 2008; Wang et al. 2008; Nagano et al. 2012; Ishikawa et al. 2015) there are no data concerning melatonin effects on sphingolipids in plants. Cho et al. (2011) showed that melatonin inhibited Sphingosine kinase 1 pathway and ROS generation in hypoxic PC-3 prostate cancer cells resulting in apoptosis of PC-3 cells.

According to our findings, melatonin protects BY-2 cells exposed to lead against cell damage and this is correlated with BI-1 protein expression that strongly increase tobacco cell viability under heavy metal stress conditions. This is the first report concerning melatonin influence on BI-1 regulation in plants, thus the mechanisms of this effect should be clarify in further studies.

Although undertaken research were carried on model suspension cells they will enable to select the proper substances to reduce environmental stresses. It has become apparent that heavy metals are becoming progressively more common as soil and water pollutants in industrialized areas. Often the contaminated soils are placed “off limits” or they are localised where they are less damaging. As an alternative, growing green plants, which take up and tolerate pollutants in the contaminated areas, are an accepted means of cleaning soil, a process referred to as phytoremediation (Reiter et al. 2015). Tan et al. (2007b) showed that melatonin addition to soil contaminated with copper was taken up by *Pisum sativum* thereby rendering it more resistant to the absorbed heavy metal thus it makes pea useful in phytoremediation; if so, it could make the plant a candidate for phytoremediation. Data of Tan et al. (2007b) showed that melatonin might be useful in enhancing the phytoremediative capacity of plants and our data point new mechanism by which this indoleamine protected plants exposed to the elevated concentrations of heavy metals.
